# Neuronal activity drives PCDH9 cleavage and nuclear translocation to coordinate structural and functional remodeling

**DOI:** 10.3389/fncel.2025.1736960

**Published:** 2026-01-28

**Authors:** Federico Miozzo, Annalaura Zambrano Avendano, Maria Giuseppa Caso, Benedetta Valentino, Shinji Hirano, Luca Murru, Edoardo Moretto, Maria Passafaro

**Affiliations:** 1 Institute of Neuroscience, CNR, Vedano Al Lambro, Italy; 2 Laboratory of Cell Biology, Faculty of Medicine, Kansai Medical University, Hirakata, Osaka, Japan; 3 NeuroMI Milan Center for Neuroscience, University of Milano-Bicocca, Milan, Italy

**Keywords:** activity-dependent signaling, cleavage, matrix metalloproteases, plasticity, Protocadherin 9, regulated intramembrane proteolysis, synapse

## Abstract

Protocadherins are key regulators of neurodevelopment and synaptic function, acting not only as adhesion molecules but also as synaptic hubs for intracellular signaling. Here, we uncover a novel activity-dependent signaling pathway for *Pcdh9*, a protocadherin linked to Autism Spectrum Disorder and Major Depressive Disorder. By combining biochemical and immunohistochemistry approaches on neuronal cultures, we show that neuronal activity triggers Matrix Metalloproteases (MMP)-dependent cleavage of PCDH9, generating a C-terminal fragment (CTF) that translocates to the nucleus. PCDH9 CTF overexpression promotes dendritic growth, increases spine density, and concomitantly strengthens excitatory synaptic transmission. These findings identify PCDH9 CTF as a novel activity-dependent signaling molecule that links synaptic activity to structural remodeling and functional modulation, suggesting a new mechanism by which synaptic activity shapes neuronal properties.

## Introduction

Protocadherin (PCDH) family comprises more than 70 transmembrane glycoproteins and represents the most extensive subgroup within the cadherin superfamily. PCDH proteins contain an N-terminal extracellular region formed by multiple extracellular cadherin (EC) domains with conserved cadherin motifs, a single transmembrane helix, and a C-terminal cytoplasmic tail. Through homophilic and heterophilic adhesive interactions mediated by their extracellular domains, PCDHs orchestrate a wide range of neurodevelopmental processes, including neurite extension, axon guidance, and the formation and maturation of synapses (Peek et al., 2017; Mancini et al., 2020). These adhesive functions are essential for establishing precise patterns of neuronal connectivity and proper wiring of neural circuits. Consistent with their central role in brain development, genetic alterations in PCDH genes have been linked to numerous neurodevelopmental and neuropsychiatric disorders (Flaherty and Maniatis, 2020; Jia and Wu, 2020).

Beyond their adhesive role, PCDHs also function as synaptic signaling hubs, modulating intracellular signaling pathways governing neuronal communication and plasticity. Through their C-terminal intracellular domains, PCDHs interact with components of multiple signaling network, including the WAVE Regulatory Complex (WRC) which controls actin cytoskeletal dynamics, as well as apoptotic and WNT signaling pathways (Pancho et al., 2020). PCDHs also influence synaptic composition by modulating phosphorylation cascades, the proteasome-ubiquitin system, and through direct interaction with GABA_A_ receptors (Yasuda et al., 2007; Tsai et al., 2012; Bassani et al., 2018). Furthermore, members of PCDH family have been shown to undergo sequential proteolytic cleavage in response to specific stimuli (Pancho et al., 2020), a mechanism also referred to as Regulated Intramembrane Proteolysis (RIP) (Lee and Ch'ng, 2020). This multi-step process is initiated by cleavage of the extracellular domain by Matrix Metalloproteases (MMPs), which in turn favors secondary or even tertiary cleavage events of the remaining membrane-bound C-terminal intracellular domain by intramembrane cleaving proteases (i-CLIPs), most notably the γ-secretase complex. Beyond serving as a degradation pathway that reduces the levels of adhesion molecules at the cell surface, RIP also generates soluble bioactive fragments: the cleaved N-terminal ectodomain, liberated into the extracellular space, can exert paracrine or autocrine effects, while the C-terminal fragment released in the cytosol may participate in downstream intracellular signaling (Lee and Ch'ng, 2020; Pancho et al., 2020).

Among the diverse PCDH family members, growing evidence has linked alterations in Protocadherin 9 (*PCDH9*) gene to neurodevelopmental and psychiatric conditions. PCDH9 has been associated with autism spectrum disorder (ASD) following the identification of copy number variations (CNV) in autistic individuals (Marshall et al., 2008; Bucan et al., 2009) and reduced *PCDH9* mRNA levels in lymphoblasts from ASD patients (Luo et al., 2012). Moreover, a meta-analysis of three genome-wide association studies identified a single nucleotide polymorphism in *PCDH9* as a risk factor for major depressive disorder (MDD) and cognitive impairment (Xiao et al., 2018). More recently, *PCDH9* has also been implicated in essential tremor (Clark et al., 2022), a common movement disorder with cognitive and neuropsychiatric components (Lombardi et al., 2001). Findings from mouse experiments further support a crucial role for *Pcdh9* in neurodevelopment and synaptic organization. *Pcdh9* knockout (KO) mice display long-term deficits in social and object recognition, hyperactivity, and impaired sensorimotor performance, accompanied by cortical thinning, reduced dendritic arborization, and increased spine density in somatosensory pyramidal neurons (Bruining et al., 2015). In the hippocampus, PCDH9 is predominantly localized at glutamatergic synapses, and its expression peaks during the first postnatal week, a critical period for synaptogenesis (Miozzo et al., 2024). *Pcdh9*-deficient neurons exhibit enlarged presynaptic terminals and postsynaptic densities in CA1, along with upregulation of synaptic genes and dysregulation of the SHANK2/CORTACTIN pathway. These transcriptional and structural alterations lead to enhanced miniature excitatory postsynaptic currents and reduced network activity, underscoring *Pcdh9* critical role in shaping excitatory synapse morphology and function (Miozzo et al., 2024). Studies using an independent *Pcdh9* KO line further revealed impaired fear extinction, potentially linked to abnormalities in the basolateral amygdala (Uemura et al., 2022).

Despite these recent advances unveiling the contribution of *Pcdh9* to neuronal and synaptic functions and behavioral traits, very little is known regarding the underlying PCDH9 molecular and signaling mechanisms. Here, we show that PCDH9 undergoes proteolytic cleavage in response to neuronal activity, generating a C-terminal fragment which migrates to the nucleus and influences neuronal morphology and function. These findings identify PCDH9 as a novel activity-dependent signaling molecule, suggesting new potential mechanisms underlying its association to neurodevelopmental and psychiatric disorders.

## Materials and methods

*Primary neuronal cultures*. Primary neurons were prepared from cortices and hippocampi of Sprague Dawley E18 rat brains as previously described (Zapata et al., 2017). For biochemical assays, neurons were plated onto 12-wells plates coated overnight with poly-L-lysine (Sigma Aldrich; 50 μg/ml in 50 mM borate buffer, pH 8.5) at 100,000 neurons per well. For immunocytochemical analyses, neurons were plated onto coverslips placed in 12-well plates and coated overnight with poly-L-lysine at 75,000 neurons per well. Neurons were grown in Neurobasal plus medium (Gibco, 21103049) supplemented with 2 % B27 plus (Gibco, 17504044), 1 % L-glutamine (Invitrogen), 1 % penicillin/streptomycin (Invitrogen), and 10 mM glutamate. The cells were maintained at 37 °C and 5 % CO_2_ in a humidified incubator (Euroclone, SafeGrow Pro). At day *in vitro* 4 (DIV4), half of the medium was replaced by fresh medium without glutamate. Then, half of the medium was changed once a week. DIV15 neurons were treated with the following reagents: NMDA (20 μM; Sigma- Aldrich), APV (100 μM; Merck), glutamate (50 μM; Tocris), bicuculline (40 μM; Sigma-Aldrich), tetrodotoxin (2 μM; Tocris), GM6001 (10 μM; Tocris). All animal procedures were approved by the Italian Ministry of Health (Ministero della Salute, Italy) and conducted in accordance with national and European regulations (Directive 2010/63/EU).

*Whole-cell lysate and nuclear-enriched fraction preparation*. DIV15 neurons were quickly washed twice with ice-cold PBS before being scraped with a cell scraper and transferred to an Eppendorf tube. For whole-cell lysate (WCL) preparation, cell suspensions were centrifuged (200 g, 10 min, 4 °C) and pellets resuspended in modified RIPA buffer (50 mM Tris-HCl, 200 mM NaCl, 1 mM EDTA, 1 % NP40, 1 % Triton X-100, pH 7.4) supplemented with Protease Inhibitor Cocktail (PIC, Roche). For nuclear-enriched fraction preparation, cell suspensions were quickly spinned down for 10 s in an Eppendorf tabletop microfuge. Cell pellets were resuspended in 700 μl of ice-cold PBS + 0.1 % NP40 (Calbiochem, CA, USA) and triturated 10 times using a P1000 micropipette. Samples were then spinned down for 10 s in an Eppendorf tabletop microfuge and the supernatant carefully discarded. The pellets containing the nuclei were resuspended in 200 μl of ice-cold PBS + 0.1 % NP40 and loaded on a 40 μm cell strainer previously humidified with PBS. Next, the samples were centrifuged as above for 10 s and the supernatants carefully discarded. The pellets containing the nuclei-enriched fraction (~ 20 μl) were dissolved in 30 μl of RIPA lysis buffer (10 mM Tris-HCl pH 8.0, 1 mM EDTA, 0.5 mM EGTA, 1 % Triton X-100, 0.1 % Sodium Deoxycholate, 0.1 % SDS, 140 mM NaCl). Protein dosage of WCL and nuclear-enriched fractions was determined using the bicinchoninic acid assay (BCA, Euroclone) prior to SDS-page and Western blotting.

*Western blots*. Samples were loaded on 9 % polyacrylamide gels and then transferred onto nitrocellulose membranes (0.22 μm, GE HealthCare). Membranes were blocked in 5 % milk and 0.1 % TBS-Tween 20 for 1 h at RT and then incubated with the primary antibodies [anti-PCDH9 homemade rat antibody directed to the C-terminal region (Asahina et al., 2012), 1:2,000; anti-GAPDH Cell Signaling (#2118), 1:10,000; anti-CTCF Cell Signaling Technology, 1:1500] in 0.1 % TBS-Tween 20 overnight at 4 °C. After washing, the blots were incubated at room temperature for 1 h with goat HRP-conjugated anti-rat (ThermoFisher Scientific, 1:5,000) or anti-rabbit (Jackson Immunoresearch, 1:20,000) antibodies in 0.1 % TBS-Tween-20. Immunoreactive bands on blots were visualized by enhanced chemiluminescence (GE HealthCare). Different exposure times were used to acquire PCDH9 FL and PCDH9 CTF band signals. Quantification of band intensity was performed with Fiji software (Schindelin et al., 2012).

*Constructs and transfections*. The DNA sequence corresponding to the cytosolic C-terminal region of human PCDH9 (UniProtKB X5D7N0, amino acids 836-1237) fused with HA tag (TAC CCA TAC GAT GTT CCA GAT TAC GCT) at the 5′ and V5 tag (GGT AAG CCT ATC CCT AAC CCT CTC CTC GGT CTC GAT TCT ACG) at the 3′ was synthetized by AZENTA Life Sciences and subcloned into *pHR-hSyn* vector to obtain the *hSYN:HA-Pcdh9CTF-V5* construct. DNA sequencing confirmed the correct sequence and position of the insert downstream to the hSYN promoter of the backbone vector. A *hSYN:GFP* plasmid expressing GFP was used to visualize transfected neurons. *hSYN:HA-Pcdh9CTF-V5* and *hSYN:GFP* plasmids were transfected into primary neurons at DIV8 using Lipofectamine 2000 (Invitrogen) according to manufacturer's instructions (ratio 1:1). Transfections were carried out in 12-well plates using 1 μg DNA per plasmid (2 μg total in case of co-transfection). Cells were fixed (immunocytochemical assays) or collected (biochemical assays) at DIV15.

*Immunocytochemistry*. Cultured neurons at DIV15 were washed in PBS and fixed in 4 % paraformaldehyde (PFA) and 10 % sucrose for 15 min at room temperature (RT). Fixative was removed by washing three times with PBS for 10 min at RT. After blocking/permeabilization in 10 % normal goat serum (NGS), 0.1 % Triton X-100, in PBS for 15 min at RT, neurons were incubated with primary antibodies (anti-HA Roche #11867423001, 1:200; anti-MAP2 Synaptic System #188004, 1:2000) in GDB 1X solution (GDB 2X: 2 % gelatin, 0.3 % Triton X-100, 0.2 M Na_2_HPO_4_ pH 7.4, 4 M NaCl) overnight at 4 °C. After three 10 min washes with high salt buffer (500 mM NaCl, 20 mM NaPO4^(2−)^ in PBS), the coverslips were incubated with secondary antibodies (anti-rabbit IgG Alexa Fluor 488-conjugated Invitrogen A11029, 1:400; anti-mouse IgG DyLight-conjugated 649 Jackson ImmunoResearch 211-492-177, 1:400) in GDB 1X solution for 1 h at RT. Neurons were washed three times with high salt buffer and incubated with DAPI (1:10,000) for 5 min at RT. After washing with PBS for 5 min at RT, coverslips were mounted with Fluoromount (Thermo Fisher Scientific).

*Image acquisition and analysis*. Images were acquired with a Zeiss LSM 800 confocal microscope (Carl Zeiss, Italy) by using 40 X/1.3 oil objective. Image stacks with approximately 6-9 images at depth interval of 0.75 μm were obtained at 1024 x 1024 pixel resolution and ~4 μs dwell time per pixel. Image analysis of PCDH9 CTF subcellular localization was performed with Fiji (Schindelin et al., 2012). DAPI and MAP2 channels were used to draw regions of interest (ROIs) corresponding to the nuclear and cell body compartments. HA fluorescent signals (mean intensity) were quantified on a single stack in which the nuclear section was wider. For morphological analysis, total dendritic length, Sholl analysis, spine density quantification, and spine classification were calculated using NeuronStudio software (Rodriguez et al., 2006). Briefly, a z-stack acquisition (40X/1.3 oil-immersion objective) was imported, calibrated in Fiji, and automatically traced by NeuronStudio software package. The total length of the dendrites was subsequently calculated. For Sholl analysis, the shell interval was set at 3 μm. In all the experiments, for each condition, a minimum of 7 neurons from 2 independent preparations was analyzed. To classify the shape of neuronal spines in culture, we adapted a previously described algorithm (Moretto et al., 2023). For the classification of spine shapes, we used the following cutoff values: aspect ratio for thin spines (AR thin (crit)) = 2.5, head-to-neck ratio (HNR (crit)) = 1.3, and head diameter (HD (crit)) = 0.250 μm, where crit indicates critical value.

*Electrophysiology*. Whole-cell patch-clamp recordings were performed at room temperature from DIV 15 primary cortical neurons in culture transfected with hSYN:V5-PCDH9 CTF-HA and hSYN:GFP, or hSYN:GFP only as a control. Neurons were perfused with an external solution containing (in mM): 138 NaCl, 4 KCl, 2 CaCl2, 1.2 MgCl2, 10 HEPES, and 10 d-glucose (pH 7.4). The external solution was supplemented with tetrodotoxin (TTX, 3 μM) and bicuculline (20 μM) to block voltage-dependent sodium channels and GABA_A_ receptors, respectively. The composition of the intracellular solution was (in mM): 126 K-gluconate, 4 NaCl, 1 EGTA, 1 MgSO4, 0.5 CaCl2, 3 ATP (magnesium salt), 0.1 GTP (sodium salt), 10 glucose, 10 HEPES-KOH (pH 7.3). Recordings were conducted with a Multiclamp 700B amplifier (Axon CNS molecular devices, USA). Pipette resistance was 2-3 MΩ and access resistance was always below 20 MΩ. Recordings were discarded if access resistance changed more than 20 % during the experiment. mEPSCs were recorded at a holding potential of −65 mV over a period of 2-5 min, filtered at 2 kHz and digitized at 20 kHz, using Clampex 10.1 software. Analysis was performed offline with Clampfit 10.1 software using a threshold crossing principle. The detection level was set at 5 pA, and raw data were visually inspected to eliminate false events.

*NLS prediction*. To search for nuclear localization signal (NLS) in PCDH9 protein sequence, the online tool NLStradamus (http://www.moseslab.csb.utoronto.ca/NLStradamus/) was used (Nguyen Ba et al., 2009). NLStradamus predicted the following NLS “KQNKKKKRKKRKSPK” in the mouse PCDH9 protein sequence (amino acids 864-878; UniProt B2RY69).

*Statistical analysis*. Statistical comparisons were performed using GraphPad Prism software (San Diego, CA). The statistical significance of differences between the two groups was calculated using unpaired *t*-test. To compare three or more groups, Kruskal-Wallis followed by Dunn's multiple comparisons test was used. For Sholl analysis, ordinary two-way ANOVA was used. Differences were considered significant at ^*^p < 0.05, ^**^p < 0.01, ^***^p < 0.001, and ^****^p < 0.0001.

## Results

### Neuronal activation triggers Matrix Metalloproteinase (MMP)-dependent PCDH9 cleavage in primary neurons

**Figure 1:**
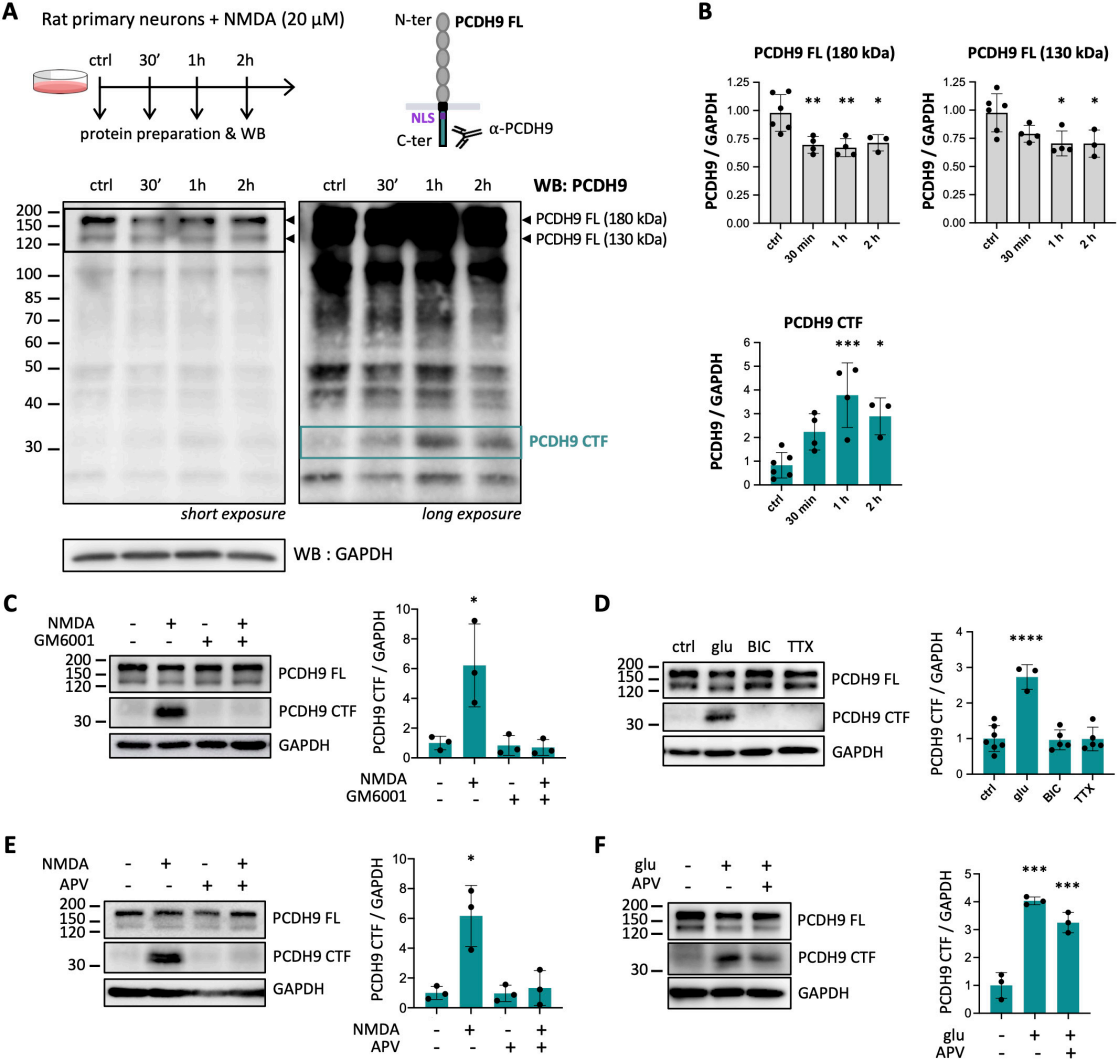
Neuronal activation triggers Matrix Metalloproteinase (MMP)-dependent PCDH9 cleavage in primary neurons. **(A)** Experimental scheme (top panel) and representative PCDH9 Western blot (bottom panels) of primary neurons treated with 20 μM NMDA for 30 min, 1 h or 2 h. At short exposures (bottom left panel), two full-length PCDH9 isoforms (PCDH FL; ~130 and ~180 kDa; indicated by arrowheads) are detected, as previously reported (Bruining et al., 2015; Miozzo et al., 2024). At longer exposure time (right bottom panel), additional lower molecular-weights bands become visible, including one above 30 kDa whose level changes with NMDA treatment (putative PCDH9 C-terminal fragment, PCDH9 CTF). GAPDH was used as a loading control. **(B)** Quantifications of PCDH9 FL and PCDH9 CTF levels from A. Protein levels were normalized to GAPDH. *n* = 3-6 independent cultures. **(C)** Representative WB and corresponding quantification of PCDH9 CTF levels in primary neurons pretreated with the MMP inhibitor GM6001 (5 h prior to NMDA exposure, 6 h total) before exposure to NMDA (1 h). *n* = 3 independent cultures. **(D)** Representative WB and corresponding quantification of PCDH9 CTF levels in primary neurons treated 1 h with glutamate (glu), bicuculline (BIC) or tetrodotoxin (TTX). *n* = 3-7 independent cultures. **(E)** Representative WB and corresponding quantification of PCDH9 CTF levels in rat primary neurons pretreated with the NMDAR antagonist APV (1 h prior to NMDA exposure, 2 h total) before exposure to NMDA (1 h). *n* = 3 independent cultures. **(F)** Representative WB and corresponding quantification of PCDH9 CTF levels in primary neurons pretreated with the NMDAR antagonist APV (1 h prior to glutamate exposure, 2 h total) before exposure to glutamate (1 h). *n* = 3 independent cultures. The bands corresponding to full-length (FL) PCDH9 are also displayed. WB quantifications are presented as protein levels normalized to GAPDH. Mean ± SD is shown. Statistical analysis was performed using Kruskal-Wallis test followed by Dunn's multiple comparisons test against untreated controls. *p < 0.05, **p < 0.01, ***p < 0.001, ****p < 0.0001.

To investigate whether PCDH9 undergoes proteolytical cleavage and intracellular signaling in response to neuronal activity, we stimulated rat primary neurons with N-Methyl-D-Aspartate (NMDA), an agonist of NMDA receptors (NMDAR), and monitored PCDH9 by Western blot (WB) over time. Since we hypothesized that NMDA might trigger the cleavage and release of PCDH9 intracellular region, we used an antibody directed against its cytoplasmic C-terminal region (Figure 1A). As previously reported (Bruining et al., 2015; Miozzo et al., 2024), two full-length (FL) PCDH9 isoforms at ~180 and ~130 kDa were detected in neurons. NMDA stimulation induced a moderate but consistent decrease of PCDH9 FL levels, suggesting potential proteolysis (Figures 1A, B, top). Upon longer membrane exposure, additional lower-molecular-weight bands became visible, most of which did not vary with treatment and were likely due to non-specific antibody recognition. Remarkably, however, a band above 30 kDa appeared specifically in response to NMDA treatment and persisted during the 2 h treatment (Figure 1A, right, and Figure 1B, bottom; PCDH9 CTF). The concomitant decrease of PCDH9 FL and emergence of this C-terminal fragment (CTF) suggests that neuronal activity might trigger PCDH9 cleavage, reducing the pool of PCDH9 FL and generating a distinct intracellular product.

To test whether PCDH9 is cleaved through Regulated Intracellular Proteolysis (RIP) initiated by Matrix Metalloproteinases (MMPs), we conducted NMDA stimulation in presence of a broad range MMP inhibitor (GM6001). Blocking MMP activity completely abolished the appearance of PCDH9 CTF, demonstrating that PCDH9 undergoes activity-induced, MMP-dependent RIP (Figure 1C). We next wanted to clarify which types of neuronal stimulation could trigger PCDH9 cleavage. Neurons were exposed to glutamate, which broadly activates excitatory transmission, tetrodotoxin (TTX), a voltage-gated sodium channel blocker that silences neuronal activity, or bicuculline, a GABA_A_ receptor antagonist that blocks inhibitory transmission (Figure 1D). As expected, glutamate triggered PCDH9 CTF generation, confirming that an excitatory input is required to induce PCDH9 cleavage, whereas TTX had no effect. Interestingly, bicuculline did not promote cleavage, suggesting that blocking inhibitory transmission alone is not sufficient to engage PCDH9 proteolytic pathway. Finally, to determine whether PCDH9 cleavage specifically relies on NMDAR activation or glutamatergic signaling more broadly, we applied the NMDAR antagonist APV. APV completely prevented NMDA-induced PCDH9 cleavage (Figure 1E), but had only a partial, non-significant effect on glutamate-treated neurons (Figure 1F). This indicates that NMDAR activation is sufficient but not necessary to trigger PCDH9 cleavage, and that additional glutamatergic receptors contribute to it. Taken together, these experiments demonstrate that glutamatergic signaling drives MMP-mediated PCDH9 cleavage, leading to the generation of PCDH9 CTF in primary neurons.

### PCDH9 CTF nuclear translocation leads to increased dendritic length and spine density and enhances synaptic transmission

**Figure 2:**
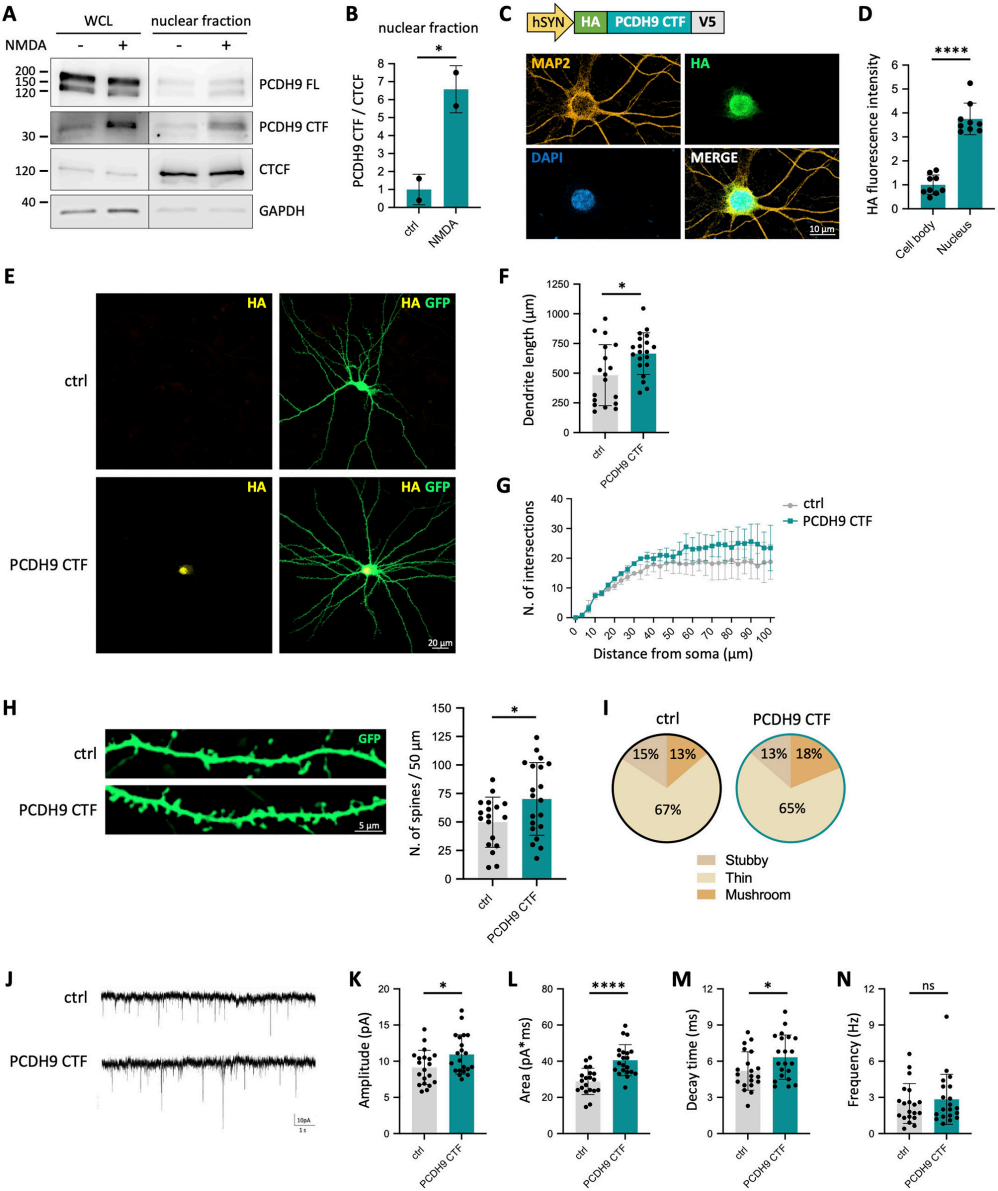
PCDH9 CTF nuclear translocation leads to increased dendritic length and spine density and enhances synaptic transmission. **(A)** Representative WB of PCDH9 FL and PCDH9 CTF in Whole Cell Lysates (WCL) and nuclear-enriched fraction after 1 h NMDA treatment of primary rat neurons. CTCF was used as nuclear marker. **(B)** Quantification of PCDH9 CTF levels in the nuclear fraction. Protein levels were normalized to CTCF. Mean ± SD is shown. *n* = 2 independentcultures. Unpaired *t*-test, *p < 0.05. **(C)** Immunostaining of primary neurons transfected with the PCDH9 CTF construct. PCDH9 CTF was visualized via anti-HA staining. Neuronal cell bodies and extensions were labeled with MAP2, and nuclei were counterstained with DAPI. **(D)** Quantification of HA fluorescence intensity in cell bodies and nuclei from C. Mean ± SD is shown. n = 9 neurons per group from 2 independent cultures. Unpaired *t*-test, *p < 0.05. **(E)** Primary neurons were co-transfected with constructs expressing PCDH9 CTF (*hSYN:HA-Pcdh9CTF-V5*) and GFP (*hSYN:GFP)* and subjected to dendritic and spine analysis. **(F)** Quantification of total dendrite length in neurons co-transfected with PCDH9 CTF and GFP vs. neurons transfected with GFP only (ctrl). Mean ± SD is shown. *n* = 18-20 neurons per group from 2 independent cultures. Unpaired *t*-test, *p < 0.05. **(G)** Sholl analysis of dendritic complexity. Mean ± SEM is shown. *n* = 31 neurons per group from 2 independent cultures. Ordinary two-way ANOVA, no significant differences. **(H)** Representative images of GFP-labeled dendrites **(left)** and quantification of spine density **(right)** in neurons expressing PCDH9 CTF and GFP vs. neurons transfected with GFP only (ctrl). Mean ± SD is shown. *n* = 18-20 neurons per group from 2 independent cultures. Unpaired *t*-test, *p < 0.05. **(I)** Pie charts illustrating the distribution of the different spine types. **(J)** Representative mEPSC traces recorded from primary neurons expressing PCDH9 CTF and GFP and control neurons transfected with GFP only (ctrl). **(K-N)** Quantification of mEPSC amplitude **(K)**, area **(L)**, decay time **(M)**, and frequency **(N)** of mEPSCs. Mean ± SD is shown. *n* = 20-21 neurons per group from 2 independent cultures. Unpaired *t*-test, *p < 0.05, ****p < 0.0001, ns, not significant.

Intracellular fragments generated by regulated proteolysis of cell adhesion molecules can migrate to distinct cellular compartments and act as signaling molecules. Therefore, we asked where PCDH9 CTF localizes upon its generation by neuronal activation. We found that the C-terminal cytoplasmic domain of PCDH9 harbors a predicted Nuclear Localization Signal (NLS; KQNKKKKRKKRKSPK), suggesting that PCDH9 CTF might be able to enter the nucleus. To test this hypothesis, we analyzed nuclear-enriched fractions from NMDA-treated cultured neurons. WB assays detected PCDH9 signal in the nucleus after NMDA stimulation (Figures 2A, B). To further confirm this finding, neurons were transfected with a construct expressing a tagged version of PCDH9 CTF. By immunostaining, we observed that PCDH9 CTF displayed a predominantly nuclear localization (Figures 2C, D). These experiments demonstrate that PCDH9 CTF can translocate to the nucleus, suggesting a potential role in gene expression regulation.

The generation of a PCDH9 CTF capable of nuclear translocation suggests that it may act as an activity-dependent signaling molecule, rather than merely representing a byproduct of PCDH9 degradation. To investigate its impact on neuronal morphology, we overexpressed exogenous PCDH9 CTF and co-transfected GFP to visualize neurons for morphological analysis (Figure 2E). PCDH9 CTF overexpression significantly increased total dendritic length compared to control neurons, indicating a potential role in promoting dendrite growth or elongation (Figure 2F). Sholl analysis showed a trend toward augmented dendritic branching complexity, although it did not reach statistical significance (Figure 2G). Examination of dendritic spines uncovered a significant increase in spine density following PCDH9 CTF overexpression, suggesting that it might enhance spine formation or maintenance (Figure 2H). No obvious change was observed in the distribution of spine types (Figure 2I). Collectively, these findings indicate that PCDH9 CTF overexpression promotes dendritic length and spine density, two key morphological features supporting neuronal connectivity, suggesting a potential role for PCDH9 CTF in modulating neuronal functional output.

The observed increased in dendritic length and spine density induced by PCDH9 CTF might underlie corresponding changes in neuronal synaptic function. To test this, we recorded mEPSCs from neurons transfected with the PCDH9 CTF construct. Our electrophysiological analysis revealed that PCDH9 CTF overexpression leads to increased mEPSC amplitude, area, and prolonged decay time, indicative of stronger synaptic input (Figures 2J–N). Altogether, these experiments indicate that PCDH9 CTF not only shapes neuronal morphology but also enhances synaptic transmission, linking structural remodeling to functional output.

## Discussion

**Figure 3:**
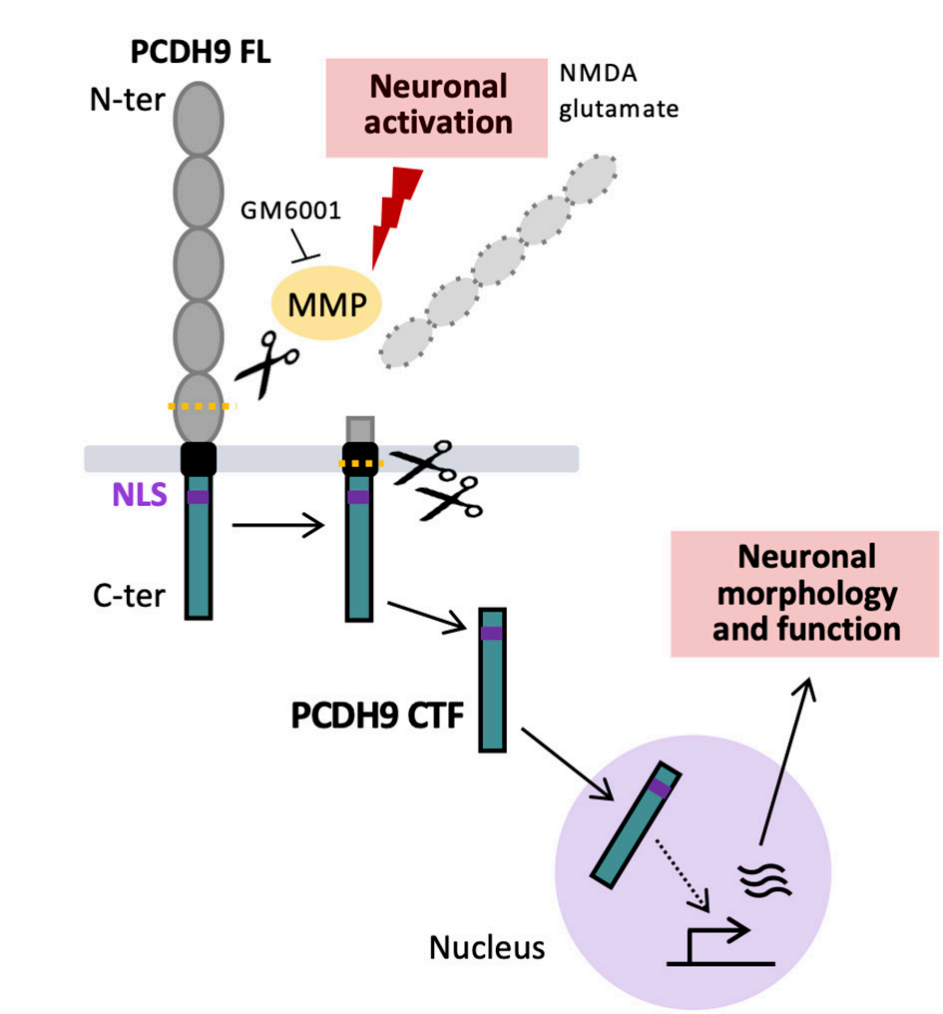
Working model. Neuronal activation triggers MMP-dependent multi-step cleavage of PCDH9, generating a PCDH9 CTF that is released in the cytoplasm. PCDH9 CTF translocates to the nucleus and modulates neuronal morphology and electrophysiological properties, possibly through gene expression regulation.

Our study demonstrates that neuronal activation triggers proteolytic cleavage of the protocadherin PCDH9, generating a C-terminal fragment that translocates to the nucleus and modulates neuronal morphology and function (Figure 3). These findings identify PCDH9 as an activity-dependent signaling molecule and uncover a novel pathway through which synaptic activity shapes neuronal properties.

Proteolytic processing in response to extracellular stimuli has been reported for several protocadherins, including γ-PCDH (Haas et al., 2005; Hambsch et al., 2005), α-PCDH (Bonn et al., 2007; Emond and Jontes, 2008), FAT1 (Magg et al., 2005), PCDH12 (Bouillot et al., 2011), and PCDH19 (Gerosa et al., 2022), often resulting in nuclear translocation of the corresponding PCDH CTF. Our biochemical and immunocytochemical analysis extend this mechanism to neuronal PCDH9, demonstrating its activity-dependent, MMP-mediated cleavage, and the capacity of the resulting PCDH9 CTF to enter the nucleus, possibly via an importin-dependent process (Lu et al., 2021) facilitated by its NLS. Collectively, these findings support the notion that proteolytical processing and CTF nuclear translocation might represent a conserved mechanism among Protocadherins, enabling the conversion of extracellular cues into adaptive nuclear responses.

One of the most significant findings of our study is that expression of exogenous PCDH9 CTF alone is sufficient to induce marked morphological and functional changes in neurons, including increased dendritic length, higher spine density, and enhanced synaptic transmission. Interestingly, NMDA bath stimulation typically elicits Long Term Depression (LTD)-like responses, such as spine loss or reduction in mEPSC frequency (Carroll et al., 1999; Henson et al., 2017). In this context, the NMDA-induced PCDH9 CTF signaling might play a homeostatic function in counteracting such changes by supporting synaptic maintenance, similar to other activity-dependent proteolytic pathways of cell adhesion molecules that promote dendritic spine development (Tian et al., 2007).

Given its pronounced nuclear localization, we hypothesize that PCDH9 CTF might regulate neuronal phenotypes by modulating gene expression. Although PCDH9 CTF does not contain any predicted DNA- or histone-binding domain and is hence unlikely to bind directly to the chromatin, it could influence transcription through interactions with nuclear regulatory complexes. Supporting this idea, recent evidence in gastric cancer cells shows that PCDH9 C-terminal region translocates to the nucleus and associates with DNA methyltransferase 1 (DNMT1), enhancing its catalytic activity and thereby repressing gene expression (Zhang et al., 2024). Interestingly, *Pcdh9* deletion leads to a moderate but widespread upregulation of synaptic genes in hippocampal neurons *in vivo* (Miozzo et al., 2024). Therefore, we could speculate that the activity-dependent PCDH9 CTF nuclear pathway described here, potentially through a PCDH9 CTF-DNMT1 interaction, could contribute to the negative regulation of synaptic gene expression in hippocampal neurons. Future research will determine whether PCDH9 can engage the DNA methylation machinery or other chromatin-associated complexes to regulate gene expression in the brain.

The functional relevance of PCDH9 nuclear signaling *in vivo* remains to be determined. *Pcdh9* deletion leads to structural alterations in mouse brain neurons, such as reduced dendritic arborization and increased spine density in somatosensory pyramidal neurons (Bruining et al., 2015), and enlarged pre- and postsynaptic compartments in CA1 neurons (Miozzo et al., 2024). Considering our findings that PCDH9 CTF overexpression influences neuronal morphology, we hypothesize that PCDH9 CTF nuclear pathway could partly underlie the neuronal phenotypes observed in *Pcdh9* KO mice. However, ablation of the *Pcdh9* gene simultaneously abolishes both its membrane adhesive functions and its intracellular signaling, making it difficult to disentangle their respective phenotypic outcomes. Generating PCDH9 mutants that selectively disrupt either MMP-dependent cleavage or CTF nuclear entry, while preserving PCDH9 adhesive function, will be instrumental to distinguish PCDH9 synaptic and nuclear roles, and determine the relative contribution of each to PCDH9-dependent phenotypes.

This study presents some limitations. Although the NMDA concentration used here (20 μM) is generally well tolerated by primary neurons and can stimulate pro-survival pathways (Soriano et al., 2006; Zhou et al., 2013), we cannot exclude a minor degree of cell toxicity, despite the absence of overt morphological changes after 1-2 h of treatment.

Moreover, we cannot exclude a contribution of glial cells to the generation of PCDH9 CTF since they also express *Pcdh9* and NMDAR (Single Cell Portal, Broad Institute). However, primary neuronal cultures typically contain a small proportion of glial cells, which in turn express NMDAR at much lower levels than neurons. As such, we expect NMDA-induced PCDH9 cleavage to occur mainly in neurons. Finally, while PCDH9 CTF overexpression experiments were instrumental to uncover the types and directionality of neuronal processes influenced by this fragment, exogenous PCDH9 CTF levels likely exceed physiological amounts. As a result, the observed phenotypes may be amplified and not fully mirror the physiological contribution of endogenous PCDH9 CTF.

Activity-dependent gene expression converts transient synaptic activity into lasting neuronal changes, driving transcriptional programs that coordinate synapse and circuit maturation during neurodevelopment, and underlie synaptic plasticity in the adult brain (Yap and Greenberg, 2018; Sugo et al., 2025). Compelling evidence links ASD to mutations in activity-dependent transcriptional pathways which control synaptic function, and in regulatory regions driving activity-induced transcription (Ebert and Greenberg, 2013; Yap and Greenberg, 2018). Within this framework, our identification of an activity-dependent PCDH9 nuclear pathway suggests a novel potential mechanism for fine tuning activity-dependent gene expression, whose dysregulation might contribute to the association between *PCDH9* genetic alterations and ASD. In conclusion, our findings provide new insights into the molecular mechanisms of a synaptic protein involved in diverse neurological disorders, extending beyond its established adhesive function. Notably, the activity-dependent generation of PCDH9 CTF suggests a role in synaptic plasticity in the mature brain, distinct from its previously described neurodevelopmental functions. Further mechanistic insights on the exact regulation of PCDH9 multi-step cleavage, nuclear translocation and nuclear functions will be critical for understanding *PCDH9* involvement in brain disorders and open new perspectives for therapeutic interventions.

## Data Availability

The original contributions presented in the study are included in the article/supplementary material, further inquiries can be directed to the corresponding author/s.
